# Long-term Prognosis of Athletes With Patellar Tendinopathy Receiving Physical Therapy: Patient-Reported Outcomes at 5-Year Follow-up

**DOI:** 10.1177/03635465251336466

**Published:** 2025-05-12

**Authors:** Jie Deng, Jelle J. Oosterhof, Denise Eygendaal, Stephan J. Breda, Edwin H.G. Oei, Robert-Jan de Vos

**Affiliations:** † Department of Orthopedics and Sports Medicine, Erasmus MC University Medical Center, Rotterdam, South Holland, Netherlands; ‡ Department of Radiology and Nuclear Medicine, Erasmus MC University Medical Center, Rotterdam, South Holland, Netherlands; § Department of Sports Medicine, Haaglanden MC, Leidschendam, South Holland, Netherlands; ‖ Department of Radiology, AZ Turnhout, Turnhout, Belgium; Investigation performed at Erasmus MC University Medical Center, Rotterdam, Netherlands

**Keywords:** knee injury, loading, recovery, treatment

## Abstract

**Background:**

Patellar tendinopathy (PT) is a highly prevalent injury among jumping athletes. The long-term prognosis of athletes with PT following physical therapy is unknown.

**Purpose:**

To assess self-perceived recovery rate and the 5-year change in pain levels, disability, and sports participation, and to explore the prognostic factors associated with self-perceived recovery.

**Study Design:**

Cohort study; Level of evidence, 3.

**Methods:**

Athletes with PT who were previously enrolled in a randomized trial and received education, load management advice, and exercise therapy instructions at baseline were eligible. An online questionnaire was sent 5 years after inclusion. Self-perceived recovery was assessed by a dichotomized 7-point global rating of change (recovery was defined as “significantly improved” to “completely recovered”). Pain levels during sports (0-10 points) and disability assessed by the Victorian Institute of Sport Assessment–Patellar (VISA-P) score were recorded at baseline and 5 years. Sports participation was categorized into return to performance, return to sport, return to participation, and quitting sports. Nonparametric tests were performed to compare scores at baseline and 5 years. Logistic regression models were used to identify prognostic factors.

**Results:**

Of 76 eligible participants, 58 (76%) responded (mean age, 30 years [SD, 4 years]; 28% female). At a mean follow-up of 5 years, 76% of participants felt recovered. Pain levels during sports (median, 7 points [IQR, 7-8 points] to 2 points [IQR, 1-4 points]) and VISA-P score (median, 57 [IQR, 45-66] to 82 [IQR, 74-97] points) significantly improved from baseline to 5 years (all *P* < .001). In total, 41 participants (71%) returned to their desired sports (68% to performance and 32% below preinjury level), 12 participants (21%) returned to participation in other sports, and 5 (9%) completely ceased sports participation. Participants who felt unrecovered had higher levels of pain and disability and lower return to performance (all *P* < .05). No prognostic factors were identified that were associated with self-perceived recovery.

**Conclusion:**

Athletes with PT after physical therapy can expect a generally acceptable long-term prognosis. However, almost one-quarter did not feel recovered and perceived worse patient-reported outcomes. Clinicians treating athletes with PT may use these findings to estimate the average prognosis.

Patellar tendinopathy (PT) is a common injury among athletes performing jumping sports,^20^ with a prevalence of 21% in basketball players and 25% in volleyball players.^25^ This condition is characterized by pain at the inferior pole of the patella^20^ and affects work capacity, sports performance,^10^ and quality of life.^31^ Exercise-based strategies have become the first-line treatment, with various therapeutic modalities as complementary options.^21,23^

The overall prognosis of PT in athletes is still uncertain. Limited small-scale studies have reported a generally poor prognosis, with 53% of male athletes ceasing their sports career after 15 years^17^ and 19% of competitive volleyball players retiring.^36^ In contrast, another study found a more optimistic outcome, with 57% of male athletes returning to their preinjury sport at 3 to 4 years.^1^ However, 2 of these studies were limited to including male players,^1,17^ while another focusing specifically on volleyball players,^36^ and their reported outcome measures did not align with current guidelines.^12,35^ Most of these studies also reflect the average PT course under clinical approaches from >10 to 20 years ago. Given the changes in PT management^6,13,23^ and the development of more reliable outcome measurement instruments for tendinopathies under relevant core domains,^12,35^ it is crucial to update and evaluate the prognosis of PT in the context of current standard care, especially with a larger sample size. Such information is essential, as it provides realistic estimates for future outcomes of PT,^14^ facilitating patient education and decision-making. Additionally, prognostic factors for the long-term course of PT remain largely unknown, which could help identify potential targets for new interventions and guidance in patient-specific treatment algorithms.^28^

Therefore, we conducted this prospective study to estimate 5-year patient-reported outcomes in athletes with symptomatic PT following education, load management advice, and exercise-based approaches. The primary aim of this study was to assess self-perceived recovery rate, change in pain levels, disability and quality of life, and sports participation. A secondary aim was to explore the prognostic factors of self-perceived recovery.

## Methods

### Study Design and Participants

This prospective cohort study is the 5-year follow-up of a previous randomized controlled trial (RCT)^5^ that compared the effectiveness between progressive tendon-loading exercises and eccentric exercises in athletes with PT (detailed exercise protocols were published in the previous work^5^). This trial was prospectively registered for short-term follow-up (6 months) on ClinicalTrial.gov (identification No. NCT02938143). The current observational study was approved by the ethics committee of Erasmus MC University Medical Center (MEC-2016-500). Because the follow-up was long-term, we expected no ongoing treatment effect of the different exercise programs implemented as part of the original trial. Also, we expected that other treatments might have been provided to some participants. For these reasons, we considered it reasonable to combine the 2 groups into 1 cohort for this longer-term analysis. All participants who volunteered for the 5-year follow-up provided digital informed consent before participation. We adhered to the STROBE (Strengthening the Reporting of Observational Studies in Epidemiology) guidelines^37^ for reporting observational studies and to the ICON (International Consensus Statement for Tendinopathy)^30^ minimum reporting standards for tendinopathy.

The original trial enrolled 76 athletes at Erasmus MC University Medical Center between 2017 and 2019, aged 18 to 35 years. Participants performed their desired sports at least 3 times per week before the injury and were diagnosed with symptomatic PT confirmed by the following criteria: patellar tendon pain increasing with activity, recognizable tenderness on patellar tendon palpation, and patellar tendon changes on ultrasound and/or increased vascularity on power Doppler ultrasound. Demographic data (eg, sex, age, body mass index [BMI], and symptom duration) and several physical tests (eg, quadriceps strength) were collected at baseline,^5,11^ and current height and weight were also collected at the 5-year follow-up. For this follow-up study, we attempted to consecutively contact all athletes by email or telephone from 2022 to 2024, which was 5 years after their start of enrollment. For those who agreed to participate and provided digital informed consent, the questionnaire was distributed via a link by email.

### Outcome Measures

All participants completed an online questionnaire with questions that can be found in Appendix A (available in the online version of this article).

#### Self-Perceived Recovery

Participants were asked about their self-perceived recovery compared with the start of the trial. This outcome was designed using the global rating of change (GROC) with a 7-point Likert scale according to the most recent guideline in tendinopathy,^12,35^ ranging from 3 (completely recovered) to −3 (worse than ever). The responses were dichotomized, with recovery defined as “significantly improved” to “completely recovered,” whereas those rated as “slightly improved” to “worse than ever” were deemed to have not recovered.^19,33^

#### Pain Levels, Disability, and Quality of Life

The pain levels during activities of daily living (ADL) and the most recent sports activity were measured on a scale of 0 to 10 points, with 0 representing no pain and 10 representing severe pain. Complete relief from pain was defined as a pain level equal to 0 at the 5-year follow-up. To assess disability and quality of life, the Victorian Institute of Sport Assessment–Patellar (VISA-P) scale^38^ and the European Quality of Life–3 Dimensions (EQ-5D-3L) index^34^ were used, respectively. The percentage of participants who achieved the known minimal clinically important difference (MCID) for these scores was reported. The MCID for the change of pain levels (>1.2 points^7^) and VISA-P score (>13 points^15^) was used for this purpose. All scales were administered in their Dutch versions and were measured at baseline (start of enrollment) and the 5-year follow-up.

#### Sports Participation

The preinjury level of sports activity was recorded at baseline (Table 1). Participants were instructed to complete their current sports participation (type, duration, and frequency per week) in the 5-year follow-up digital questionnaire (Appendix A, available online). The physical demands of sports were categorized from high to low intensity, as previously defined^4^: jumping, hard pivoting, and cutting (eg, basketball, volleyball, football); running, twisting, and turning (eg, racquet sports, baseball, hockey); and no running, twisting, or jumping (eg, cycling, swimming).

**Table 1:** Baseline Characteristic of Participants (N = 58)*^a^*

Characteristic	Value
Demographics	
Age, y	24 (4)
Male sex	42 (72)
Height, cm	184 (10)
Weight, kg	82 (12)
BMI, kg/m^2^	24 (3)
Tendinopathy descriptors	
Symptom duration, wk	106 [52-247]
VISA-P score (0-100 points)	57 [45-66]
VAS score during a single-leg decline squat (0-10 points)	5 [3-7]
Imaging for assisting diagnosis	
Use of ultrasound and MRI	58 (100)
Physical test results	
Quadriceps muscle strength, N/kg	5 (1)
Sports characteristics before the injury	
Physical demands of desired sport*^b^*	
Jumping, hard pivoting, cutting (eg, basketball, volleyball)	53 (91)
Running, twisting, turning (eg, racquet sports)	5 (9)
No running, twisting, jumping (eg, cycling, swimming)	00 (0)
Frequency of sports activity, d/wk	3 [3-3]
Adjustment of desired sport at baseline	
Stopped	21 (36)
Decreased	22 (38)
Equal	15 (26)

a Data are presented as n (%), mean (SD), or median [IQR]. BMI, body mass index; d/wk, days per week; N/kg, Newtons per kilogram; MRI, magnetic resonance imaging; VAS, visual analog scale; VISA-P, Victorian Institute of Sport Assessment–Patellar; wk, weeks; y, years.

b The category was based on a previous study.^4^

Sports participation was categorized into (1) return to performance (participating in the desired sport at or above the preinjury level), (2) return to sport (participating in the desired sport below the preinjury level), (3) return to participation (changed from the desired sport to another sport), and (4) completely quit sports participation, according to the most recent consensus.^2^ The rate of return to desired sport was defined as the proportion of participants who resumed their preinjury sports at 5 years, regardless of performance level (categories 1 and 2 combined). Participants who returned to participation or completely stopped sports participation were subsequently instructed to choose the most crucial reason for this change, including PT symptoms, fear of reinjury of PT, and non–PT-related reasons (eg, change of interest, lack of time).

#### Health Care Consumption

Participants were instructed to select which treatments they received between the end of the trial and the 5-year time point using designed checkboxes and open-ended questions (Appendix A, available online).

#### Proposed Prognostic Factors

Predefined baseline variables were used to evaluate the prognostic value of self-perceived recovery. All potential prognostic factors were assessed at baseline. These were sex, age, BMI, symptom duration (weeks), VISA-P score, and quadriceps muscle strength (the maximal isometric voluntary contraction measured by a handheld dynamometer in N/kg).^5,11^

### Statistical Analysis

We performed the analyses based on the complete case, presenting descriptive statistics as numbers and percentages for categorical data (eg, self-perceived recovery and sports participation) and central tendency and dispersion for continuous data. Normally distributed data, inspected using a histogram and Shapiro-Wilk test, are given as mean and standard deviation; otherwise, median and interquartile range (IQR) are presented. We decided to perform a subgroup analysis of participants with self-perceived recovery and nonrecovery for the patient-reported outcomes (pain levels, VISA-P score, EQ-5D-3L, and sports participation). Between-group comparisons were assessed using *t* tests for normalized continuous data. For nonnormalized data, the Wilcoxon signed-rank test was performed for paired data, and the Mann-Whitney *U* test was used for non-paired data. The Fisher exact test was used for categorical comparison. Logistic regression models were used to assess the prognostic value of predefined baseline variables for self-perceived unrecovered events. These were done using initial univariable analyses, and variables with a *P* value <.15 were included^27^ in the multivariable model, adjusted for treatment allocation^24^ from the previous RCT.^5^ All calculations were performed using RStudio Version 2023.12.0 (Posit). *P* values <.05 were considered statistically significant.

## Results

**Figure 1.**
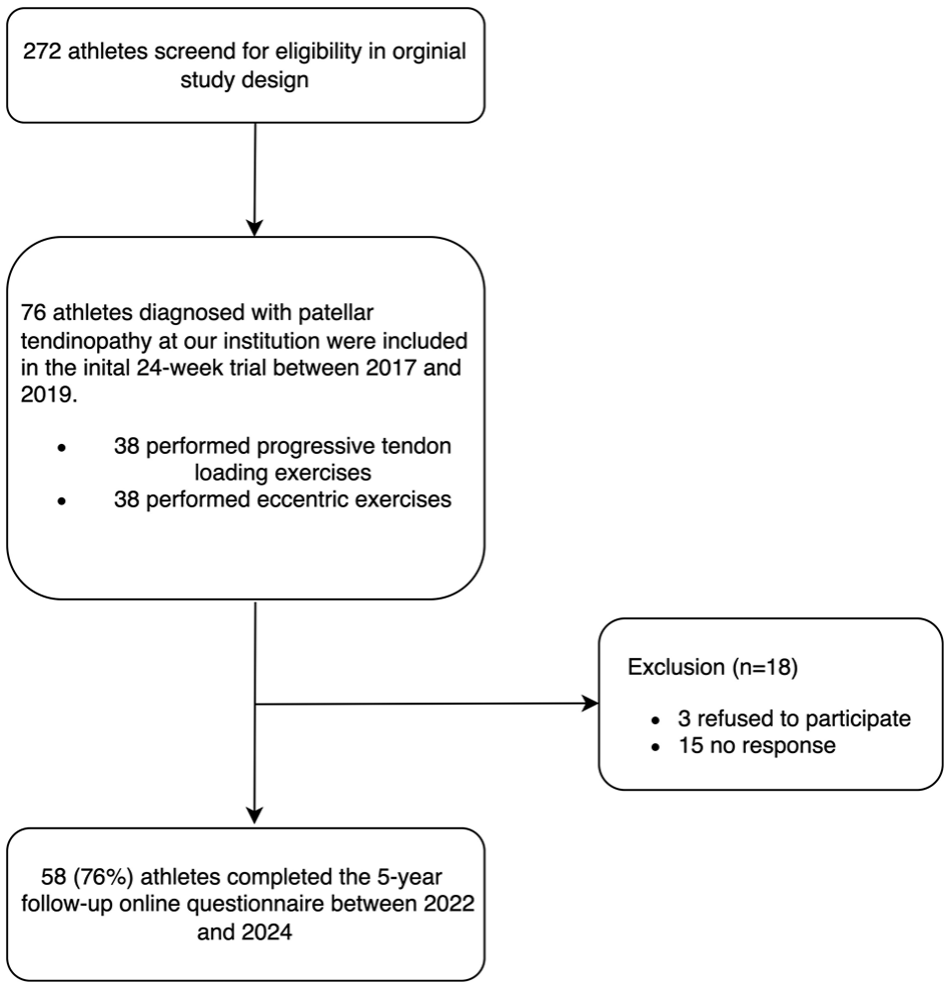
Flowchart of study design.

Of the 76 athletes included at baseline, 58 (76%) completed the online questionnaire at a mean follow-up of 5 years (SD, 0.2 years). At follow-up, the mean age was 30 years (SD, 4 years), 28% of the athletes were female, and the mean height and weight were 185 cm (SD, 10 cm) and 84 kg (SD, 14 kg), respectively. The baseline characteristics for these participants at the start of the JUMPER study are shown in Table 1. Of the 18 nonparticipants, 3 (17%) refused to participate in the follow-up assessment, and 15 (83%) did not respond to telephone or 2 email reminders within 4 weeks (Figure 1). There was no significant difference in baseline variables between responders and nonresponders (Appendix Table B1, available online).

Most participants (95%) received or applied nonoperative treatment approaches between the end of the trial (6-month follow-up) and the 5-year follow-up. One participant received patellar tendon debridement surgery at 2.5 years (Table 2).

**Table 2:** Treatment Approaches Participants Underwent Between 6-Month and 5-Year Follow-up (N = 58)

Treatment	No. (%)
Rest	37 (64)
Adjustment of sports activities	40 (69)
Stretching exercises	25 (43)
Strengthening exercises	42 (72)
Foot orthoses	12 (21)
Knee brace	6 (10)
Patellar strap	15 (26)
Medical taping	9 (16)
Manual therapy	1 (2)
Shock-wave therapy	7 (12)
Ultrasound therapy	4 (7)
Dry needling	6 (10)
Medication (paracetamol and/or anti-inflammatory agents)	8 (14)
Surgery (patellar tendon debridement)	1 (2)

### Self-Perceived Recovery

Of the 58 participants, 44 (76%) were defined as recovered at 5 years, with 16 (36%) stating complete recovery and 28 (64%) reporting significant improvement. Fourteen participants (24%) did not feel recovered. Among those who were categorized as nonrecovered, none reported a worse condition compared with baseline (Table 3).

**Table 3:** Self-Perceived Recovery Using the GROC With 7-Point Likert Scale (N = 58)*^a^*

Item	No. (%)
Recovered	44 (76)
Completely recovered	16 (28)
Significantly improved	28 (48)
Nonrecovered	14 (24)
Slightly improved	8 (14)
Remained the same	6 (10)
Slightly worsened	00 (0)
Significantly worsened	00 (0)
Worse than ever	00 (0)

a Global rating of change (GROC) was dichotomized into “recovered” and “nonrecovered.”

### Change in Pain Levels, Disability, and Quality of Life

There was a significant improvement in the median scores for pain levels, disability level, and quality of life from baseline to the 5-year follow-up (all *P* < .001). The proportion of participants who achieved the MCID at 5 years was 61% for pain levels during ADL, 67% for pain levels during recent sports activity, and 71% for the VISA-P score. Additionally, at the 5-year follow-up, 21 participants (36%) reported complete relief from pain during ADL, and 13 (25%) were completely pain-free during sports activity (Table 4).

**Table 4:** Comparison of Pain Levels, Disability, and Quality of Life Between Values at Baseline and 5 Years*^a^*

Variable (points)	Baseline, Median [IQR]	5 y, Median [IQR] or No. (%)	*P* Value	MCID Achieved, %
Pain levels during ADL (0-10)	4 [3-6]	1 [0-3]	<.001	61
Complete relief from pain (0)		21 (36)		
Pain levels during recent sports activity (0-10)	7 [7-8]	2 [1-4]^*b*^	<.001	67
Complete relief from pain (0)		13 (25)^*b*^		
VISA-P score (0-100)	57 [45-66]	82 [74-97]	<.001	71
EQ-5D-3L index (0-1)	0.84 [0.81-0.84]	0.98 [0.90-0.99]	<.001	NA

a ADL, activities of daily living; EQ-5D-3L, European Quality of Life–3 Dimensions; MCID, minimal clinically important difference; NA, nonapplicable; VAS, visual analog scale; VISA-P, Victorian Institute of Sport Assessment–Patellar.

b Because 5 participants quit sports at 5 years, 53 rated their pain levels.

In addition, there were significant differences in the medians of these change scores between those who felt recovered and nonrecovered (Appendix Table B2, available online).

### Sports Participation

**Figure 2.**
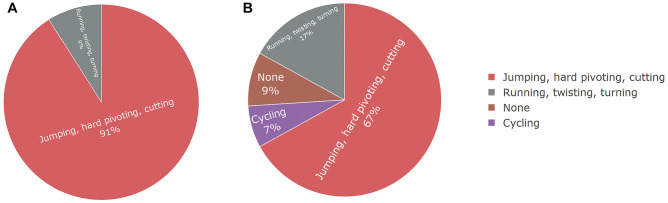
Types of sports participation at (A) preinjury and (B) 5-year follow-up based on the level of physical demand.

The types of sports participation at preinjury and 5-year follow-up are shown in Figure 2 based on the level of physical demand.

A total of 41 participants (71%) returned to their desired sport. Of these, 28 (68%) performed at or above their preinjury level, while 13 (32%) performed below their preinjury level. Twelve athletes (21%) returned to participation by modifying their desired sports into other sports, whereas 5 (9%) completely ceased sports participation. The reasons for modifying or discontinuing sports participation were both PT related and non–PT related (Table 5).

**Table 5:** Sports Participation at 5-Year Follow-up*^a^*

Sports Participation	Value
How many times per week do you currently participate in sport?	3 [2-3]
How many hours do you practice your sport on average per week?	2 [2-4]
Category of sports participation	
Return to performance	28 (48)
Return to sport	13 (22)
Return to participation	12 (21)
Because of PT symptoms	3 (25)
Because of fear or reinjury of the patellar tendon	2 (17)
Because of non–PT-related reasons	7 (58)
Completely stopped sports participation	5 (9)
Because of PT symptoms	3 (60)
Because of non–PT-related reasons	2 (40)

a Data are presented as n (%) or median [IQR]. PT, patellar tendinopathy.

The frequency of sports activity per week at the 5-year follow-up (median 3 times per week [IQR 2-3 times per week]) was significantly lower than the preinjury level (median 3 times per week [IQR 3-3 times per week]) (*P* < .001). Specifically, 58% of participants reported a decrease in sports frequency, 6% reported an increase, and 36% maintained the same frequency.

### Subgroup Analysis of Nonrecovered Participants

In the subgroup analysis (Table 6), participants who felt unrecovered had greater impairments in pain levels, disability, quality of life, and sports participation at 5 years compared with those who felt recovered (all *P* < .05).

**Table 6:** Subgroup Analysis of Participants With Self-Perceived Recovery and Nonrecovery for the Patient-Reported Outcomes at 5 Years*^a^*

Outcome at 5 years	Recovery (n = 44)	Nonrecovery (n = 14)	*P* Value
Pain levels during ADL	1 [0-2]	4 [2-5]	<.001
Pain levels during sports*^b^*	1 [0-3]	6 [5-7]	<.001
VISA-P score	90 [75-87]	73 [63-78]	<.001
EQ-5D-3L index	0.987 [0.984-0.987]	0.898 [0.846-0.984]	.002
Sports participation			.027
Return to performance	25 (57)	3 (21)	
Return to sport	6 (14)	7 (50)	
Return to participation	9 (20)	3 (21)	
Quit sports	4 (9)	1 (7)	
Frequency of sports activity	3 [3-4]	3 [2-3]	<.001
Duration of sports activity	2 [2-4]	3 [2-3]	.926

a Data are presented as n (%) or median [IQR]. ADL, activities of daily living; EQ-5D-3L, European Quality of Life–3 Dimensions; VISA-P, Victorian Institute of Sport Assessment–Patellar.

b Because 5 participants quit sports at 5 years, 53 rated their pain levels.

### Prognostic Factors

In the univariable analyses, higher BMI and VISA-P scores at baseline were sufficiently associated with a higher probability of self-perceived recovery and were therefore entered into the multivariable model. However, neither was statistically significant in the multivariable analysis (Table 7).

**Table 7:** Univariable and Multivariable Models Evaluating the Association Between Baseline Factors and Self-Perceived Recovery Status*^a^*

	Univariable		Multivariable	
	OR (95% CI)	*P* Value*^b^*	OR (95% CI)	*P* Value
Female sex	1.24 (0.40-7.61)	.556		
Age, y	1.09 (0.93-1.30)	.308		
BMI, kg/m^2^	1.26 (0.97-1.74)	.108	1.27 (0.97-1.77)	.112
Symptom duration, wk	1.00 (0.99-1.00)	.200		
VISA-P score (0-100 points)	1.03 (0.99-1.09)	.148	1.03 (0.99-1.09)	.158
Quadriceps muscle strength, N/kg	0.95 (0.53-1.72)	.862		

a BMI, body mass index; y, years; wk, weeks; N/kg, Newtons per kilogram; VISA-P, Victorian Institute of Sport Assessment–Patellar.

b Factors with *P* values <.15 in the univariable model were subsequently entered into the multivariable analysis, adjusted for treatment allocation (in our case, 2 variables were selected for inclusion in the multivariable analysis).

## Discussion

Our study provides long-term data for athletes with PT following current quality of care using comprehensive patient-reported outcomes. We observed a self-perceived recovery rate of 76% and return to desired sport rate of 71%, along with sustained improvements in pain levels, disability, and quality of life. However, only 25% of participants reported complete relief from pain during sports at the 5-year follow-up, and mild yet persistent symptoms were observed in most cases. Additionally, more than half of the athletes reported reduced frequency in sports participation. Participants who felt unrecovered showed higher levels of pain and disability and lower quality of life and were less likely to return to performance compared with those who experienced recovery. No prognostic factors for self-perceived recovery were identified.

### Comparison of Current Findings With the Literature

#### Prognosis Based on Sports Participation

To our knowledge, the long-term prognosis of PT based on sports participation has only been assessed in a limited number of small-scale studies. One earlier cohort study reported generally poor outcomes, with a sports cessation rate of 53% (9 of 17 athletes)^17^ after 15 years. In contrast, our study found a substantially lower sports cessation rate of 9% (of these, 60% were because of PT symptoms), suggesting an improved prognosis based on perceived functional status. This overall improvement could be explained by advances in the management of PT over time.^23^ Compared with this previous cohort, most of the athletes in our study followed a 24-week evidence-based^21^ exercise regimen, such as progressive tendon-loading exercises or eccentric exercise therapy, and often supplemented with active or passive therapeutic modalities. These approaches likely contributed to a higher return to sport rate. This speculation was also supported by a recent cohort study, which showed that 57% (16 of 28 male athletes) returned to their preinjury sport at 3 to 4 years after a structured 12-week exercise treatment.^1^ However, caution is warranted when interpreting these differences, given the variations in outcome measures and study populations. Previous studies often used dichotomous outcomes (eg, quit or not), whereas our study adhered to the current consensus guidelines^2^ by categorizing sports participation into 4 distinct levels (returning to performance, sport, or participation and quitting). This approach allowed us to provide a more nuanced and comprehensive assessment of sports participation, reducing the potential overestimation associated with dichotomous measures, particularly in studies with small sample sizes. Furthermore, differences in cohort characteristics may account for some observed discrepancies. For example, 2 previous studies focused exclusively on male athletes,^1,17^ and another study was limited to volleyball players.^36^ In contrast, our study included female participants and a broader range of sports. Additionally, the longer follow-up in previous studies may have contributed to the higher sports cessation rate.

#### Prognosis Based on GROC

To our knowledge, no study on PT has reported a long-term prognosis based on the outcome measured by GROC. However, a favorable prognosis—79% of patients felt recovered based on a dichotomized GROC—has been reported after 1 year of education and exercise therapy in patients with gluteal tendinopathy.^22^ This instrument has been recognized as one of the core outcome measures in tendinopathy research.^12,35^ Because prognosis is a major concern for patients,^14^ integrating patient-centered outcome measures such as a GROC is highly relevant. The GROC allows individuals to focus on the concerns most relevant to them,^16^ providing a holistic view when patients rate their course of conditions. Consistent with findings in other musculoskeletal disease studies.^8,16^ we found that participants who felt they had recovered had larger improvements in pain levels, disability, and quality of life (Appendix Table B2, available online), indicating that the GROC can reflect a multifaceted perspective on perceived change. Moreover, we observed that a higher percentage of participants felt recovered (76%) compared with those achieving the MCID for domain-specific measures (eg, 61%-67% for pain levels during ADL and sports and 71% for VISA-P score). This finding is not surprising as GROC has been reported to contain additional constructs,^16^ including aspects like change in emotional well-being,^18^ that are not fully reflected in these specific clinical instruments. One notable finding from our study is that the majority of participants felt recovered despite the persistence of pain (25% reported complete relief from pain). It seems plausible that athletes with PT may care more about overall improvement during recovery than about the complete absence of pain.

### Clinical Implications

This observation has important clinical implications. Specifically, healthcare providers could use these data to facilitate patient education and decision-making while administering treatment.

Healthcare professionals should communicate this generally positive prognosis to patients, underscoring that most athletes with PT experience recovery and return to their preinjury sport under current standard treatment approaches. It is important to balance this optimism with realistic expectations about the possibility of mild but long-standing symptoms. By addressing this, patients may remain motivated and engaged in their rehabilitation programs rather than being frustrated if full symptom resolution is not achieved.

It is also crucial to recognize that approximately one-quarter of athletes in our study did not feel recovered, highlighting the need for a more comprehensive rehabilitation approach. Beyond current therapies, these patients may benefit from an extensive intervention that integrates effective pain management,^23^ functional maintenance, and the provision of psychological or social support.^32^ Such approaches may also help athletes overcome the fear of reinjury, facilitating a full return to sport. More invasive treatments, like surgery,^3^ could be considered for refractory conditions.

### Strengths and Limitations

This is the first and largest study to report 5-year follow-up data in athletes with PT using a broad spectrum of outcome measures aligned with the core domains established for tendinopathy research.^12,35^ Additionally, we were able to contact a large number of participants during follow-up, and we did not identify relevant differences between responders and nonresponders, which increases the likelihood that responders were representative of the included group at baseline. The relationship between GROC and outcome changes assessed using serial measurement instruments was examined. Thus, the potential recall bias of using GROC was reduced as much as possible.

However, several limitations of this study should also be acknowledged. The generalizability of our findings may be limited, as participants were drawn from a randomized trial with strict inclusion criteria, potentially excluding individuals relevant to the broader prognosis.^26,29^ In addition, our findings could only reflect the average prognosis of people managed with the aforementioned treatment modalities. These results may not necessarily apply to settings with other quality of care. For example, the exercise regimens used in the original trial may differ from those typically applied in general clinical practice. Another limitation of this study is the difference in pain measurement tools used at baseline (visual analog scale) and during follow-up (numeric rating scale via digital questionnaire). However, both utilized a 0 to 10 scale and are generally considered interchangeable in tendinopathy practice.^9^ Furthermore, we were unable to further explore the variability of the prognosis based on different treatment strategies, such as different exercise programs (progressive tendon loading exercises vs eccentric exercises), nonoperative versus operative (only 1 participant received surgery), and passive treatments versus injections, due to the limited sample size and the application of co-interventions in most participants. We did not ask participants about the time frames when they returned to sport. Considering the long-standing nature of PT, only reporting this outcome as a binary aspect does not fully capture the course of this outcome. Finally, we did not identify any relevant prognostic factors due to the limited statistical power, which may hamper us in identifying potential targets to improve prognosis or treatment response. Larger cohort studies with extended follow-up periods are warranted. We used the categorization of sports participation to present the prognosis in sports activity. Future research could address this using real-time GPS-based data to better capture changes in activity levels and accurately observe the influence of PT.

## Conclusion

Athletes with PT have a generally acceptable long-term prognosis after physical therapy, with the majority receiving multiple interventions. Approximately three-quarters of the patients felt recovered and returned to their desired sports at 5 years of follow-up, with significant improvement in specific outcome domains. However, it should be noted that only 25% of the athletes who maintained sports participation were completely pain-free, and 58% of the cases reduced frequency in sports activity. Nearly one-quarter of participants did not feel recovered and perceived higher pain levels and disability and lower quality of life and were less likely to return to performance. No prognostic factors for recovery could be identified. Clinicians may use these findings to estimate the average prognosis when educating athletes with PT.

## Supplemental Material

sj-pdf-1-ajs-10.1177_03635465251336466 – Supplemental material for Long-term Prognosis of Athletes With Patellar Tendinopathy Receiving Physical Therapy: Patient-Reported Outcomes at 5-Year Follow-up Supplemental material, sj-pdf-1-ajs-10.1177_03635465251336466 for Long-term Prognosis of Athletes With Patellar Tendinopathy Receiving Physical Therapy: Patient-Reported Outcomes at 5-Year Follow-up by Jie Deng, Jelle J. Oosterhof, Denise Eygendaal, Stephan J. Breda, Edwin H.G. Oei and Robert-Jan de Vos in The American Journal of Sports Medicine
