# Supplementary material for: Long-term Prognosis of Athletes With Patellar Tendinopathy Receiving Physical Therapy: Patient-Reported Outcomes at 5-Year Follow-up
Source: Am J Sports Med. 2025 May 12;53(7):1568–76. doi: 10.1177/03635465251336466 (PMC12125489; doi:10.1177/03635465251336466)
Supplement: sj-pdf-1-ajs-10.1177_03635465251336466 – Supplemental material for Long-term Prognosis of Athletes With Patellar Tendinopathy Receiving Physical Therapy: Patient-Reported Outcomes at 5-Year Follow-up [file sj-pdf-1-ajs-10.1177_03635465251336466.pdf]

**Long-Term Prognosis of Athletes with Patellar Tendinopathy Receiving Physical Therapy: Patient-Reported Outcomes at 5-Year Follow-up**

Appendix A online questionnaire

Appendix B:

Table B1 Comparison in baseline variables between responders and non-responders

Table B2 Comparison of Improvement in Pain Level, Disability and Quality of Life in Participants with Self-Perceived Recovery and Non-Recovery

**Self-rating recovery**

Question: Compared to your complaints at the start of your participation in the JUMPER study, how much has your condition recovered?

1. Completely recovered
2. Significantly improved
3. Slightly improved
4. Remained the same
5. Slightly worsened
6. Significantly worsened
7. Worse than ever

**Sports participation**

Question: Are you currently practicing your desired sport that you performed before suffering your patellar tendon injury?

1. Yes
2. No

Question: are you practicing your desired sport from before your patellar tendon injury at your former level?

1. Yes
2. No, I am performing lower than my preinjury level
3. No, I am performing better than my preinjury level

Question: If you did not return to your desired sports, did you modify your activities or make a transition to playing other sports?

1. Yes
2. No

Question: If you modified or changed your desired sports, what's your current sport?

Question: Are you currently not participating in any sports?

1. Yes
2. No

Question: Did you modify your current sports activities or stop sports participation because of the following reasons? (please rank)

1. Patellar tendon injury
2. Fear of re-injury of my patellar tendon
3. Change of interest
4. Lack of time
5. COVID-19 restrictions
6. Other injuries
7. Others

Question: How often do you practice your sport per week?

Question: How many hours do you practice your sport on average per week?

Question: Indicate the extent of pain from your patellar tendon injury during activities of daily living (ADL) and your recent sport activity respectively in the past week . Choose the applicable answer.

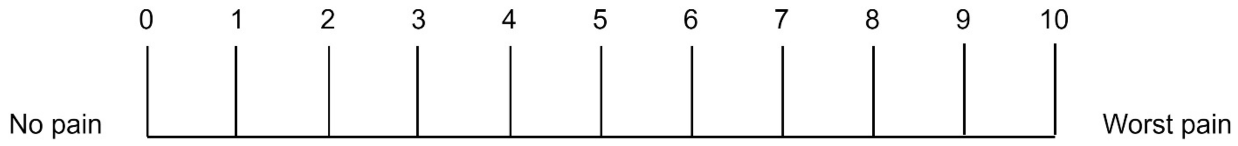


**Health care consumption**

Question: This question is about the treatments you (possibly) applied for your patellar tendon injury between 6 months after the start of your participation in the JUMPER study and now (total of 54 months). Which treatments were applied to promote the recovery of the patellar tendon injury? (multiple answers possible)

1. None
2. Rest
3. Adjusting sports activities
4. Strengthening the muscles of the lower limbs
5. Stretching the muscles of the lower limbs
6. Foot orthoses (orthotic insoles, adjusted shoes)
7. Knee brace
8. Patellar strap
9. Medical taping
10. Massage therapy

11. Manual therapy
12. Therapeutic ultrasound
13. Shock-wave therapy
14. Platelet-rich Plasma (PRP)
15. Corticosteroid injection
16. Prolotherapy
17. Dry needling
18. Percutaneous needle electrolysis
19. Medication (use of paracetamol, anti-inflammatory agents)
20. Surgery
21. Others, namely:

**Validated questionnaires in the Dutch language:**

VISA-P score

EQ-5D-3L

Table B1: The comparison between responders and non-responders in baseline characteristics[a]

| Characteristics | All (n=76) | Responders (n=58) | Non-responders (n=18) | P value[b] |
|---|---|---|---|---|
| **Demographic** | | | | |
| Age, y | 25 (4) | 25 (4) | 24 (4) | 0.709 |
| Gender, male, n (%) | 58 (76) | 42 (72) | 16 (89) | 0.211 |
| Height, cm | 185 (9) | 185 (10) | 185 (8) | 0.937 |
| BMI, Kg/m$^2$ | 23.7 [22.0-25.0] | 23.5 [22.0-25.1] | 23.8 [22.4-24.8] | 0.625 |
| **Tendinopathy descriptors** | | | | |
| Symptom duration, wk | 104 [49-208] | 106 [52-247] | 75 [37-104] | 0.081 |
| Bilateral tendon involvement, n(%) | 32 (42) | 27 (47) | 5 (28) | 0.183 |
| VISA-P score, (0-100) | 55 (13) | 56 (13) | 54 (13) | 0.659 |
| VAS during a single-leg decline squat, (0-10) | 5 [3-7] | 5 [3-7] | 6 [3-6] | 0.763 |
| **Sports characteristics before injury, n(%)** | | | | |
| Sports activities by CSAS, n(%) | | | | 0.747 |
| Level 1: 4-7 d/wk | 17 (22) | 14 (24) | 3 (17) | |
| Level 2: 1-3 d/wk | 59 (78) | 44 (76) | 15 (83) | |
| **Clinical assessment** | | | | |
| Quadriceps muscle strength, N/kg | 4.7 (1.0) | 4.7 (1.0) | 4.7 (1.0) | 0.916 |
| **EQ-5D-3L index (0-1)** | 0.84 [0.81-0.84] | 0.84 [0.81-0.84] | 0.84 [0.81-0.90] | 0.486 |

[a]Data are reported mean (SD) and median [IQR]. [b]For continuous data, a t-test was used to compare the two groups. Otherwise, a Mann-Whitney U was performed. For categorical data, we used Fisher's exact test. Abbreviations: y: years; BMI: body mass index; wk: weeks; N/kg: Newton/kilogram; CSAS: Cincinnati Sports Activity scale; VAS: visual analogue scale; VISA-P: the Victorian Institute of Sport Assessment-Patellar; EQ-5D-3L: European Quality of Life-3 Dimensions.

Table B2: Comparison of improvement in pain level, disability and quality of life in participants with self-perceived recovery and non-recovery[a]

| | Recovery (n=44) | Non-recovery (n=14) | P value |
|---|---|---|---|
| Improvement in VAS during ADL | 3 [2-5] | 1 [-2-2] | .002 |
| Improvement in VAS during sports | 5 [4-7] | 2 [-1-3] | <.001 |
| Improvement in VISA-P score | 29 [20-40] | 13 [2-23] | .017 |
| Improvement in EQ-5D-3L index | 0.144 [0.057-0.176] | 0.012 [-0.016-0.093] | .003 |

[a]The values are denoted as the absolute median [interquartile range, IQR] by subtracting the value from 5 years to baseline. Abbreviations: VISA-P: the Victorian Institute of Sport Assessment-Patellar; VAS: visual analogue scale; EQ-5D-3L: European Quality of Life-3 Dimensions.
